# Mosquito sex and mycobiota contribute to fructose metabolism in the Asian tiger mosquito *Aedes albopictus*

**DOI:** 10.1186/s40168-022-01325-9

**Published:** 2022-08-30

**Authors:** Morgane Guégan, Edwige Martin, Van Tran Van, Benjamin Fel, Anne-Emmanuelle Hay, Laurent Simon, Noémie Butin, Floriant Bellvert, Feth el Zahar Haichar, Claire Valiente Moro

**Affiliations:** 1 Univ Lyon, Université Claude Bernard Lyon 1, CNRS, INRAE, VetAgro Sup, UMR Ecologie Microbienne, F-69622 Villeurbanne, France; 2 Univ Lyon, Université Claude Bernard Lyon 1, CNRS, ENTPE, UMR 5023 LEHNA, F-69622 Villeurbanne, France; 3 MetaboHUB-MetaToul, National Infrastructure of Metabolomics and Fluxomics, Toulouse, France; 4 TBI, Université de Toulouse, CNRS, INRAE, INSA, Toulouse, France; 5 INSA-Lyon, Université Claude Bernard Lyon 1, CNRS, UMR5240, Microbiologie, Adaptation, Pathogénie, Université Lyon, 10 rue Raphaël Dubois, 69622 Villeurbanne, France

**Keywords:** Mosquito, Microbiota, Fructose, Sugar assimilation, Fungi, ^13^C metabolomic, Stable isotope probing

## Abstract

**Background:**

Plant floral nectars contain natural sugars such as fructose, which are a primary energy resource for adult mosquitoes. Despite the importance of carbohydrates for mosquito metabolism, a limited knowledge is available about the pathways involved in sugar assimilation by mosquitoes and their associated microbiota. To this end, we used ^13^C-metabolomic and stable isotope probing approaches coupled to high-throughput sequencing to reveal fructose-related mosquito metabolic pathways and the dynamics of the active gut microbiota following fructose ingestion.

**Results:**

Our results revealed significant differences in metabolic pathways between males and females, highlighting different modes of central carbon metabolism regulation. Competitive and synergistic interactions of diverse fungal taxa were identified within the active mycobiota following fructose ingestion. In addition, we identified potential cross-feeding interactions between this. Interestingly, there is a strong correlation between several active fungal taxa and the presence of fructose-derived metabolites.

**Conclusions:**

Altogether, our results provide novel insights into mosquito carbohydrate metabolism and demonstrate that dietary fructose as it relates to mosquito sex is an important determinant of mosquito metabolism; our results also further highlight the key role of active mycobiota interactions in regulating the process of fructose assimilation in mosquitoes. This study opens new avenues for future research on mosquito-microbiota trophic interactions related to plant nectar-derived sugars.

**Graphical Abstract:**

Video abstract

**Supplementary Information:**

The online version contains supplementary material available at 10.1186/s40168-022-01325-9.

## Background

Causing more than 700,000 deaths annually, mosquito-borne diseases remain a great threat to public health [1]. Female mosquitoes are hematophagous and transmit pathogens through blood feeding on vertebrate hosts. Both sexes feed on different forms of plant sugar, including flower nectar, fruit juices, plant sap, and honeydew. Nectar is a sugar-rich liquid mainly composed of sucrose, fructose, and glucose [2]. These carbohydrates influence many essential functions in mosquitoes, such as survival, flight, egg production, and energy storage [3]. Recently, it was also demonstrated that dietary glucose is an important determinant of mosquito vector competency for *Plasmodium* in *Anopheles* mosquitoes, which is linked to mosquito-microbiota interactions in regulating the development of the malaria parasite [4]. Mosquito saliva contains enzymes such as α-glucosidases, maltases, and α-amylases, which are transported with the ingested nectar [5]. After ingestion, the nectar is first stored in the crop and then transported slowly into the gut [6]. In *Anopheles aquasalis*, no activity of these enzymes was detected in the crop, suggesting that sugar digestion occurs completely in the gut [7]. In order to identify novel metabolic targets for effective mosquito control strategies, it is critical to shed some light on mosquitoes sugar metabolism. The recent development of mass spectrometry-based metabolomics combined with ^15^N (^15^nitrogen) or ^13^C (^13^carbon) isotope tracing allowed high-precision dynamic studies and revealed metabolic pathways at the atomic level in mosquito samples [8]. However, such approaches focused mainly on blood metabolism [9]. Despite the increasing amount of knowledge about carbohydrate metabolism in mosquitoes, comprehensive metabolomics data are limited, especially with regard to the specificities of sugar uptake and assimilation by mosquito sex. Meanwhile, biology is undergoing a major transition considering individuals as holobionts, i.e., entities emerging from the interaction between the host and the entire associated community of microorganisms that constitute their microbiota. Thus, mosquitoes are now considered holobionts, and important roles of microorganisms have been highlighted in mosquito biology, such as reproduction, development, and pathogen interference [10–12]. As mentioned above, mosquito’s gut is an essential organ for nutrient acquisition but also the main reservoir for microbiota. However, the roles of microorganisms in carbohydrate metabolism in mosquitoes have only been marginally explored.

Most studies on mosquito microbiota have largely focused on bacteria [10, 11], and less is known about other microbes colonizing mosquitoes, especially fungi. Recent studies have prompted investigations of non-entomopathogenic fungal interactions and have highlighted the roles of fungi in different mosquito functions, such as in their life history traits, vector competence, and behavior [13]. Yeast diets are of high nutritional quality and are used as a food source to provide proteins, vitamins, and amino acids for both larvae and adult mosquitoes [14, 15]. Moreover, a recent study highlighted that fungi are able to stimulate larval growth only when fungi are alive [16]. This could explain why yeast-fed larvae develop more rapidly than non-yeast-fed larvae, as already previously observed [15, 17, 18]. Previous investigations have shown that floral nectar harbors a diverse fungal community [19], suggesting that some mosquito-associated mycobiota in adults are environmentally acquired [20]. The role of mosquito-associated microbiota in sugar digestion and how the mosquito benefits from microbiota activity have been poorly explored. Recently, we demonstrated using a stable isotope probing (SIP) approach that the fungal community colonizing the *Ae. albopictus* gut was active in fructose assimilation [12]. However, to our knowledge, no study has yet investigated the dynamics of fructose metabolism or the fungal communities involved in its assimilation in the *Ae. albopictus* gut.

The aim of this study was to (i) evaluate how mosquitoes metabolize fructose by identifying fructose-derived metabolites and test whether mosquito sex drives fructose metabolism and (ii) investigate the dynamics of the active mycobiota associated with fructose and assimilation of derived metabolites in *Ae. albopictus* gut. For this purpose, we fed female and male *Ae. albopictus* mosquitoes with a ^13^C-fructose solution at different time periods. Fructose-derived metabolites were analyzed using a ^13^C-metabolomics approach, and mycobiota dynamics were investigated by SIP coupled with metabarcoding. We identified key and active fungal taxa involved in fructose assimilation, some of which were correlated with fructose-derived metabolites. We also emphasize that mosquito sex is an important determinant of fructose metabolism and active mycobiota diversity and highlights the importance of mosquito-mycobiota interactions in carbohydrate metabolism.

## Methods

### Mosquito breeding and experimental design

**Fig. 1:**
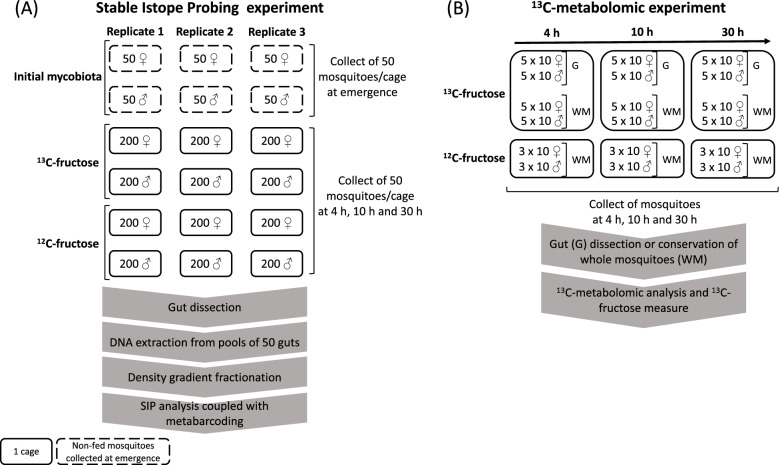
Schematic visualization of the overall experiment. **A** Experimental design of SIP experiments. The full squares represent one cage with the number of mosquitoes indicated inside. The dotted squares represent non-fed mosquitoes collected at emergence. **B** Experimental design of ^13^C-metabolomic experiments. The full squares represent one cage with the number of mosquitoes indicated inside. The number of collected mosquitoes for each type of replicate is indicated into the squares. G, guts; WM, whole mosquitoes

Eggs collected from gullies in the city of Sainte-Marie on the French Island of Reunion were used to start the *Aedes albopictus* mosquito colony. Larvae from hatched eggs were reared in dechlorinated water in plastic bowls at 25 °C with 16:8-h light:dark photoperiod and daily fed a 75/25 mixture of fish food (TetraMin®, Melle, Germany) and yeast tablet (Biover®, Nazareth, Belgium) until pupation. Adults were raised at 28 °C and 80% humidity inside climatic chambers (Panasonic MLR-352, Kadoma, Japan) under 16:8-h light:dark photoperiod and continuously fed 10% sucrose. For the SIP experiment, 2-week-old females and males were used. A total of 200 mosquitoes were randomly placed into separated cages (*n* = 12 cages, 6 for females, and 6 for males) and fed 10% ^12^C (^12^carbon)-fructose solution (control) or 10% pure (> 98% atom ^13^C) ^13^C-fructose solution (Cambridge Isotope Laboratories, Tewksbury, USA) (1:1 ratio) (Fig. 1). Samples consisted in 50 mosquitoes per cage collected after 4 h, 10 h, and 30 h of fructose feeding and stored at −20 °C (Fig. 1). High mortality of males during the experiment was prevented from having male samples after 30 h of fructose ingestion. A control corresponding to the initial gut mycobiota before feeding was performed by sampling mosquitoes directly after their emergence in adults. Three replicates (*n* = 50) per sex were constituted (Fig. 1). Mosquitoes were surface sterilized, and guts were dissected as previously described [21]. A total of 50 guts per replicate was pooled in 1× PBS (phosphate buffer saline, Life Technologies, NY, USA) and stored at −20 °C for DNA extraction.

### DNA extraction

Samples, containing 50 guts, were centrifuged at 13,200 rpm for 30 min. Pellets were resuspended in 180 μL of ATL lysis buffer (QIAGEN, Hilden, Germany) containing 0.44 μg.mL^−1^ lysozyme (Euromedex, Strasbourg, France). Genomic DNA was extracted as previously described [22] using the DNeasy Blood and Tissue kit (QIAGEN) and stored at −20 °C until further procedure. The DNA concentration was measured using the UVmc^2^ spectrophotometer (SAFAS, Monaco).

### Density gradient fractionation

Extracted DNA were fractionated by density gradient ultracentrifugation using cesium chloride (CsCl) as previously described [12]. Centrifuged fractions of approximately 340 μL were collected from top to bottom using 1 mL syringes fitted with 21-gauge needles. For each fraction, the density was measured using a 3225 refractometer (Carl Zeiss, Oberkochen, Germany), and DNA was quantified using the Quant-iT PicoGreen dsDNA Assay Kit (Life Technologies) and purified from CsCl salts with the Geneclean Turbo Kit (MP Biomedicals, Santa Ana, USA). For each gradient, two fraction representatives of labeled DNA (heavy-DNA fractions) and two representatives of unlabeled DNA (light-DNA fractions) were selected.

### Determination of ^13^C-DNA enrichment

^13^C enrichment of DNA (total, heavy- and light-DNA fractions) was determined using an elemental analyzer coupled with an isotope ratio mass spectrometer (EA/IRMS) (VarioPyroCube and Isoprime 100, Elementar Analysensysteme GmbH, Langenselbold, Germany) as previously described by Haichar et al. (2007) [23]. 13C/12C ratios of DNA were expressed as *δ* in ‰ relative to V-PDB.

### Fructose assay by GC-MS

Fructose levels were measured in female and male guts fed a solution containing 10% of full ^13^C fructose (> 98% atom ^13^C). Females and males were collected after 4 h, 10 h, and 30 h following fructose ingestion (Fig. 1). Guts were dissected on ice; 10 guts for each replicate (*n* = 5) were pooled in tubes previously weighed containing ultrapure water (Life Technologies) and 1 mm diameter beads and conserved at −80 °C until further analyses. Samples were dipped in liquid nitrogen and grinded during 1 min at 30 Hz using a bead mill (QIAGEN, TissueLyserII). Solvents are of analytical grade and provided by Fisher Scientific. Each tube was filled with 1 mL of extraction solvent consisting in acetonitrile:methanol:water (2:2:1, v:v:v) with 125 mM formic acid, vortexed and left 1 h at −20 °C. The tubes were then centrifuged 5 min at 14,000 rpm (Eppendorf, centrifuge 5424) and dried using a vacuum concentrator (Labconco, acid-resistant CentriVap concentrator and −105 °C CentriVap cold trap) before storage at −80 °C. These extracts were used for ^13^C-metabolomics and fructose assays. For fructose assays, dried extracts and a dye set of ^13^C marked D-fructose standard solutions were derivatized as previously described [24]. Within 24 h after derivatization, gas chromatography-mass spectrometry (GC/MS) analyses were performed as previously described [12].

### ^13^C-metabolomics analyses of the key metabolites and pathways related to fructose assimilation

Dry extracts of whole mosquitoes and guts obtained as described above were resuspended in 100 μl of water for ^13^C metabolomics. Isotopic profiles analysis of central metabolites was performed by high-performance anion exchange chromatography (Dionex ICS-5000+ system, Sunnyvale, USA) coupled with a LTQ Orbitrap Velos mass spectrometer (Thermo Fisher Scientific, Waltham, MA, USA) equipped with a heated probe electrospray ionization (ESI) probe. Samples were analyzed in the negative FTMS mode at a resolution of 60,000 (at m/z 400) with the following source parameters: capillary temperature 350 °C, source heater temperature 350 °C, sheath gas flow rate was 50, auxiliary gas flow rate 5, S-Lens RF level 60%, and source voltage 2.7 kV. The injection volume was 15 μL. Samples were injected on a Dionex IonPac AS11 column (250 × 2 mm) equipped with a Dionex AG11 guard column (50 × 2 mm). Mobile phase was composed of a KOH gradient which varied as follows: 0 min 0.5, 1 min 0.5, 9.5 min 4.1, 14.6 min 4.1, 24 min 9.65, 31.1 min 90, 43 min 90, 43.1 min 0.5, and 50 min 0.5. Isotopic profiles analysis of amino acids was performed by liquid chromatography (Vanquish UHPLC system, Thermo Fisher Scientific, Waltham, MA, USA) coupled with an Orbitrap Q Exactive+ mass spectrometer (Thermo Fisher Scientific) equipped with a heated ESI probe. Mass spectrometry analyses were performed in the positive FTMS mode at a resolution of 70,000 (at m/z 400) with the following source parameters: capillary temperature 320 °C, source heater temperature 300 °C, sheath gas flow rate 40, auxiliary gas flow rate 10, S-Lens RF level 40%, and source voltage 5 kV. Samples were injected on a Supelco HS F5 Discovery column (150 mm × 2.1 mm; 5-μm particle size) equipped with a Supelco HS F5 guard column (20 mm × 2.1 mm; 5-μm particle size). Solvent A was 0.1% formic acid in H_2_O, and solvent B was 0.1% formic acid in acetonitrile at a flow rate of 250 μL.min^−1^. The solvent B was varied as follows: 0 min: 2%, 2 min: 2%, 10 min: 5%, 16 min: 35%, 20 min: 100%, 24 min: 100%, 24.1 min: 2%, and 30 min: 2%. The volume of the injection was 5 μL.

Isotopic cluster for all compounds was determined by extracting and integrating the exact mass of all ^13^C isotopologues with emzed® (http://emzed.ethz.ch/) [25]. Isotopologue distributions and mean ^13^C enrichment were quantified from mass fractions after correction for the presence of all naturally occurring isotopes and isotopic purity of the tracer (> 98% atom ^13^C) using IsoCor v2.0.4 [26], which ensures accurate correction of high-resolution isotopic data. Inter-group comparisons were performed using a Wilcoxon rank-sum test of nonparametric data using sex and time as factors.

### Illumina sequencing and data analysis

Fungal ITS (internal transcribed spacer) regions were amplified in triplicate from light- and heavy-DNA fractions using the primers gITS7 (5′-GTGAATCATCGARTCTTTG-3′) and ITS4 (5′-TCCTCCGCTTATTGATATGC-3′) combined with Illumina adapters and an 8 bp multiplex barcode as described in Luis et al. (2019) [27]. For each sample, the three PCR products were pooled. Samples were purified using Agencourt AMPure XP PCR Purification kit with a 0.8× ratio (Beckman Coulter) and quantified with the Quant-iT Picogreen dsDNA Assay Kit (Life Technologies). Illumina MiSeq sequencing (2 × 300 bp paired-end reads) was performed by Biofidal (Vaulx-en-Velin, France). A total of 8,530,998 reads was obtained and demultiplexed (Biofidal). The FROGS pipeline described by Escudie et al. (2016) [28] with parameters analysis described in Guégan et al. (2018) [21] was used to control the quality and analyze the sequences. Taxonomic affiliation was carried out with the mothur pipeline [29] at 80% minimum bootstrap using a naïve Bayesian classifier [30] with the ITS UNITE database [31]. Contaminants were filtered out using negative control (blank extraction and PCR). Operational taxonomic units (OTUs) were removed if they were detected in the negative control sample and their relative abundance was not at least 10 times greater than that observed in the negative control. Normalization was performed at 3248 sequences. A total of 571 OTUs were obtained. Data and statistical analyses were performed using R software version 4.0.2 [32] with the packages phyloseq 1.32.0 [33], vegan 2.5-7 [34], ggplot2 3.3.4 [35], ape 5.4 [36], ggrepel 0.9.1 [37], plyr 1.8.6 [38], and dplyr 1.0.7 [39]. Correlation analyses between enriched fungi and fructose-derived metabolites were performed using the R package corrplot 0.89 [40]. All analysis codes and parameters were deposited in the following site: https://github.com/morganeguegan/Mosquito-sex-and-active-mycobiota-are-important-contributors-of-fructose-metabolism-in-the-mosquito.git.

### Enrichment factor (EF)

To identify fungal taxa involved in ^13^C-fructose assimilation over time, an enrichment factor (EF) was calculated for each OTU as previously described [41]:

$$EF={}^{13}{\mathrm{C}}_{\mathrm{heavy}}/{}^{13}{\mathrm{C}}_{\mathrm{light}}-{}^{12}{\mathrm{C}}_{\mathrm{heavy}}/{}^{12}{\mathrm{C}}_{\mathrm{light}}$$

where ^13^C_heavy_ and ^13^C_light_ represented the relative abundances of OTUs in heavy- and light-DNA fractions of mosquitoes fed ^13^C-fructose, and ^12^C_heavy_ and ^12^C_light_ represented the relative abundances of heavy- and light-DNA fractions of mosquitoes fed ^12^C-fructose. An OTU was considered enriched in ^13^C when the EF was > 0.5, and the relative abundance was > 0.1% of the total of sequences.

## Results

### ^13^C-fructose uptake and its metabolic pathways

**Fig. 2:**
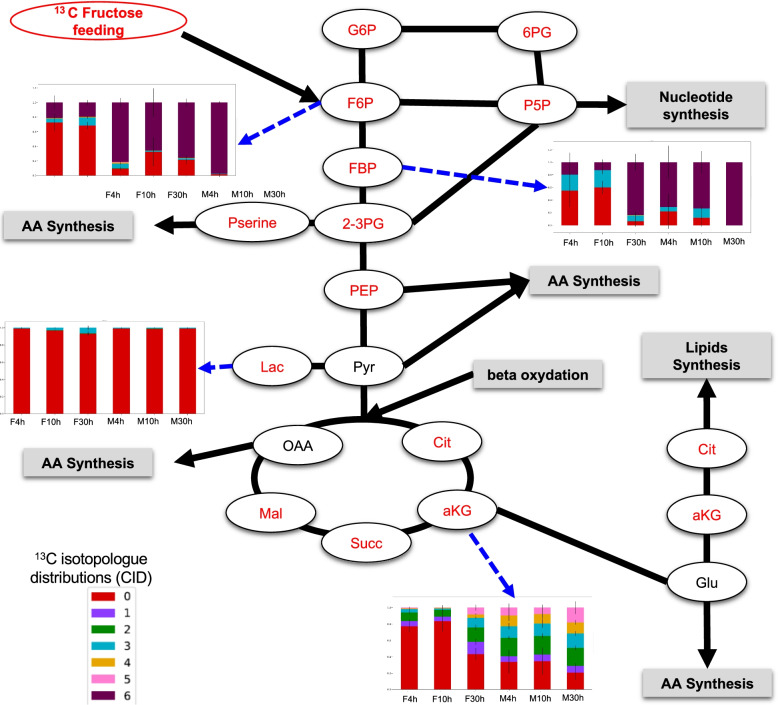
Gut detection of ^13^C enrichment in central metabolism after mosquito feeding with ^13^C-fructose. Labeled compounds detected with the analytical method are indicated in red. The bar charts represent the ^13^C isotopologue distributions (CID). Results are presented as the mean ± SD of three to five independent biological replicates per mosquito sex and time following fructose ingestion

**Fig. 3:**
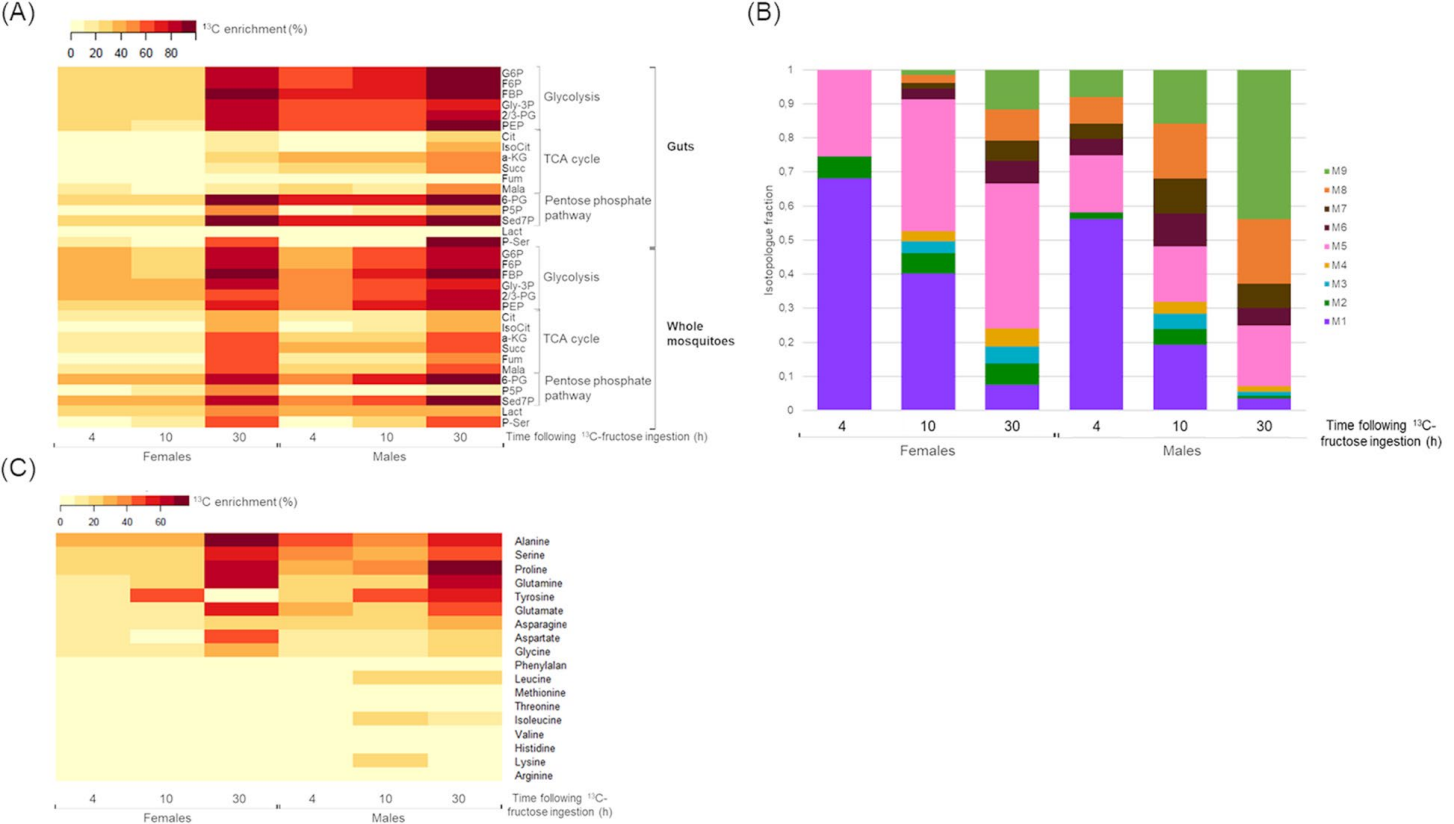
**A** Heatmap indicating time-dependent ^13^C enrichment of metabolites in guts and whole mosquitoes following ^13^C-fructose ingestion. Main pathways are indicated on the right as well as metabolites (F6P, fructose-6-phosphate; FBP, fructose-1,6-bisphosphate; Gly-3P, glyceraldehyde-3-phosphate; 2/3-PG, 2/3-phosphoglyce frate; PEP, phosphoenolpyruvate; Lact, lactate; Cit, citrate; IsoCit, isocitrate; a-KG, α-ketoglutarate; Succ, succinate; Fum, fumarate; Mala, malate; G6P, glucose-6-phosphate; 6-PG, 6-phosphogluconate; P5P, pyridoxal-5-phosphate; Sed7P, sedoheptulose-7-phosphate; P-Ser, phosphoserine; Pyr, pyruvate; OAA, oxaloacetate). **B** Time-dependent ^13^C-isotopologue distributions of UMP in male and female whole mosquitoes following ^13^C-fructose ingestion. **C** Heatmap indicating time-dependent ^13^C enrichment of amino acids in whole mosquitoes following ^13^C-fructose ingestion. Results are representative of three biological replicates per mosquito sex and time following fructose ingestion

The ^13^C-fructose levels measured in female guts were stable over time (Table S1). On the contrary, males exhibited decreasing levels of ^13^C-fructose in their guts over time (Table S1). The ^13^C isotopic profiling of a subset of fructose pathway-specific metabolites was analyzed in the guts by LC-HRMS (Fig. 2) to determine ^13^C enrichment and ^13^C isotopologue distributions (CIDs). At 4 h, ^13^C incorporation into intermediates of the glycolysis pathway fructose-6-phosphate (F6P), fructose-1,6-biphosphate (FBP), glyceraldehyde-3-phosphate (Gly3P), and phosphoenolpyruvate (PEP) was observed in both male and female mosquitoes (Fig. 3A). For each sex, the level of ^13^C enrichment was similar between 4 h and 10 h and exhibited increased levels at 30 h (Fig. 3A). However, at earlier time points (4 h and 10 h), differences were observed according to sex, with lower values measured in females (Fig. 3A); this suggests probable differences in the metabolic pathways implemented by both sexes for fructose metabolism. The analysis of CIDs showed significative differences between males and females (Table S2), indicating that females incorporated ^13^C-fructose more slowly than males and possessed different isotopologue distributions. For instance, higher M3/M6 ratios were observed in females for F6P (*p*-value = 7.93 × 10^−3^, *p*-value = 1.58 × 10^−2^, *p*-value = 2.85 × 10^−2^ at 4 h, 10 h, and 30 h, respectively) and FBP (*p*-value = 7.93 × 10^−3^, *p*-value = 1.58 × 10^−2^, *p*-value = 2.1 × 10^−2^ at 4 h, 10 h, and 30 h, respectively), thus indicating a higher metabolic mixing in females compared to the direct contribution of fructose (Fig. 2, Table S2). Regarding a-KG, an earlier and increased incorporation of this metabolite was observed in males compared to females (Fig. 2). Moreover, some metabolites (e.g., 6-PG (6-phosphogluconate), F6P, FBP) existed mainly in the full-labeled form of the molecule, indicating that these metabolites were synthesized from the fully marked fructose (Fig. 2). Furthermore, an increase of ^13^C enrichment of intermediates of the tricarboxylic acid (TCA) cycle (citrate (Cit) and α-ketoglutarate (a-KG)) and the pentose phosphate pathway (glucose-6-phosphate (G6P) and sedoheptulose-7-phosphate (Sed7P) was observed in both sexes (Fig. 3A). Interestingly, in whole male mosquitoes, the results showed a constant ^13^C-incorporation of lactate (a metabolite synthesized at the end of glycolysis) with a higher enrichment than that observed in females at early time points (Fig. 2, Table S2). However, a slight increase was observed from 10 h in females (Fig. 2). ^13^C enrichment of metabolites after ^13^C-fructose incorporation was more important in whole mosquitoes than in the guts suggesting that fructose can be metabolized anywhere in the body (Fig. 3A). Nucleotide levels were below detection threshold in the guts. However, some nucleotides were detected in whole mosquitoes, and only uridine monophosphate (UMP) and guanosine monophosphate (GMP) were labeled in both sexes (Fig. 3B). The CID indicated that the proportion of the predominant isotopologues form of UMP was different between females and males (Fig. 3B). In females, the proportion of M5 isotopologue of UMP, corresponding to the complete (5-carbon) labelling of ribose in purine nucleotides, increased over time, indicating that fructose is the major source of ribose 5-phosphate for nucleotide synthesis. Conversely, in males, the proportion of M9 isotopologue increased over time, meaning that the full molecule was labeled (i.e., ribose part and uridine part). These results indicate that nucleoside biosynthesis from fructose is different (pool and/or flux) between males and females (Fig. 3B). The amino acid pool was weak and close to the detection limit in the guts. Of those that were detected (aspartate, glutamate, leucine, methionine, proline, and tyrosine), no ^13^C enrichment was detected (Fig. 3C). Conversely, in whole mosquitoes, 18 amino acids were detected, and half of them were ^13^C-enriched (alanine, asparagine, aspartate, glutamate, glutamine, glycine, proline, serine, and tyrosine). More specifically, glycine, proline, and serine were labeled at 10 h, while alanine, aspartate, and glutamate were labeled at 30 h (Fig. 3C). Taken together, these results suggest that there are different sugar assimilation kinetics and metabolic pathways in the two sexes.

### Assimilation of ^13^C fructose

**Fig. 4:**
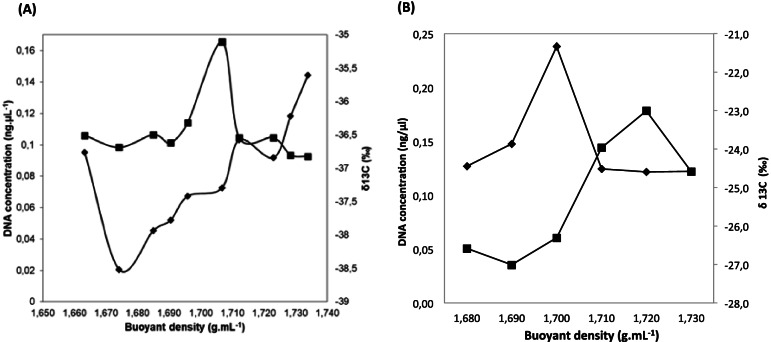
Example of incorporation of ^13^C isotope into DNA in male (**A**) and female (**B**) mosquitoes after 4 h of ^13^C-fructose ingestion. The DNA concentration of the different gradient fractions separated by CsCl gradient centrifugation was evaluated by fluorometry (■), and the amount of ^13^C was represented by the δ^13^C value measured by IRMS (♦)

After 4 h of ^13^C-fructose ingestion, the isotopic ratio, expressed as δ^13^C value, the total DNA from pooled female guts was approximately −12.55‰, confirming the ^13^C incorporation into the DNA of the mosquito and its associated microbiota. ^13^C incorporation was then evaluated along the density gradient after fractionation by ultracentrifugation. An example of the distribution of δ13C along the different DNA fractions of the density gradient is shown for one male and one female at 4 h (Fig. 4). A summary of the average values of δ13C calculated from the three replicates of each sex at each time point is available in Table S3. δ^13^C increased along the gradient from −38.51‰ at the lowest density (1.66 g.mL^−1^) to −35.60‰ at the highest density (1.73 g.mL^−1^). A peak of ^13^C enrichment of −36.57‰ was observed at a density of 1.71 g.mL^−1^, corresponding to heavy DNA and was correlated with an increase in the DNA amount (Fig. 4). Similar profiles were obtained from the other samples (data not shown). These results allowed us to select two fractions between densities of 1.68 and 1.69 g.mL^−1^ corresponding to the light DNA and two fractions between densities of 1.71 and 1.72 g.mL^−1^ corresponding to the heavy DNA for each gradient.

### Gut mycobiota dynamics after ^13^C-fructose assimilation

**Fig. 5:**
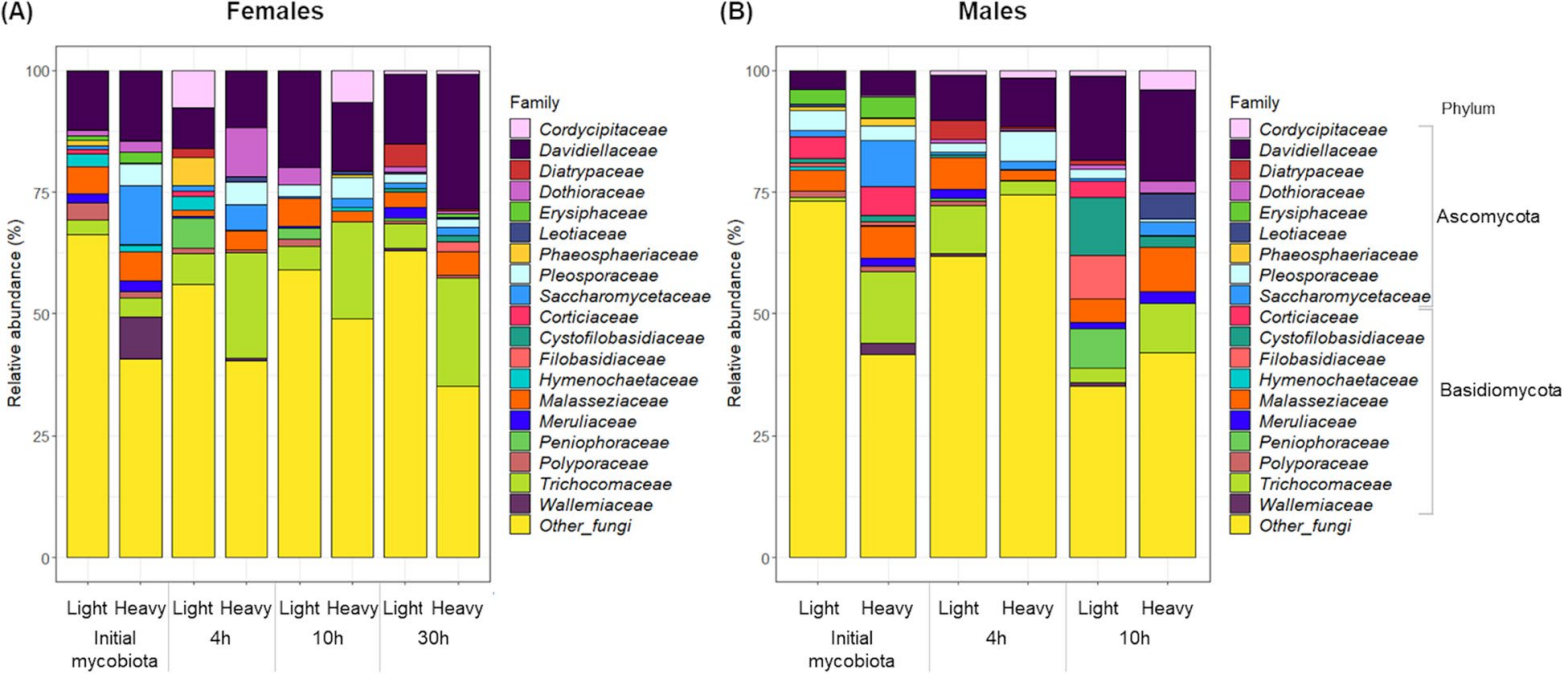
Relative abundances of fungal families in **A** females and **B** males. The relative abundances are represented in light and heavy DNA fractions from gut mosquitoes collected after mosquito emergence (initial mycobiota) and after 4 h, 10 h, and 30 h following ^13^C-fructose ingestion. Results are representative of three biological replicates per mosquito sex and time following fructose ingestion

**Fig. 6:**
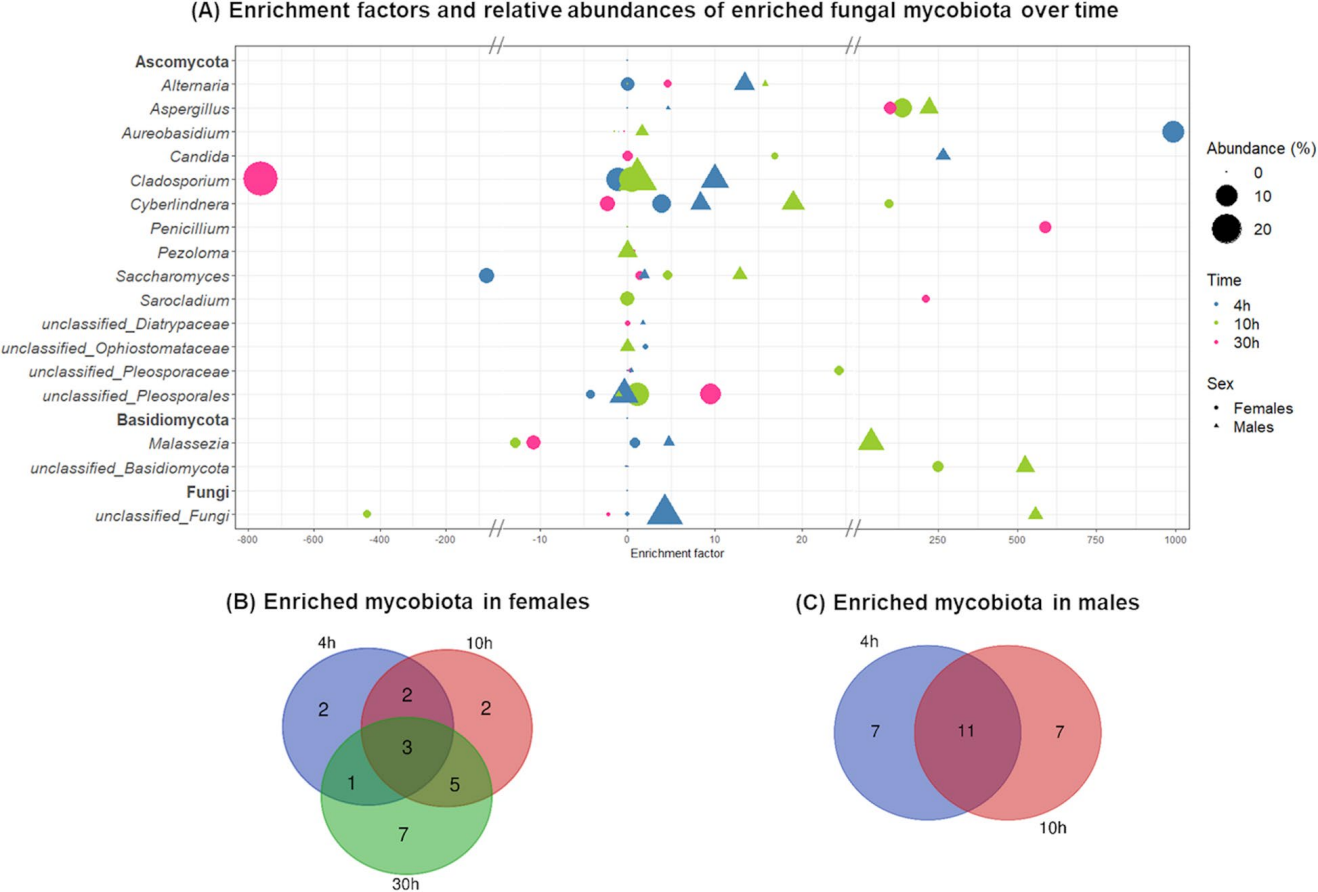
**A**^13^C-labeled fungi identified after 4 h, 10 h, and 30 h of fructose ingestion. An enrichment factor (EF) was calculated by comparing heavy and light DNA fractions from the ^12^C- and ^13^C-fructose-fed mosquitoes for each OTU. Only fungal candidates with an EF > 0.5 and a relative abundance > 0.1% are represented in these parameters, meaning they are enriched in ^13^C. Females and males are represented in circle and triangle, respectively. Mosquitoes fed during 4 h, 10 h, and 30 h are represented in blue, green, and pink, respectively. The size of the triangles and circles is proportional to the relative abundance of each fungal genera identified. Venn diagram representing the number of OTUs ^13^C enriched common or specific at each time of fructose ingestion in **B** females and **C** males. Results are representative of three biological replicates per mosquito sex and time following fructose ingestion

**Fig. 7.**
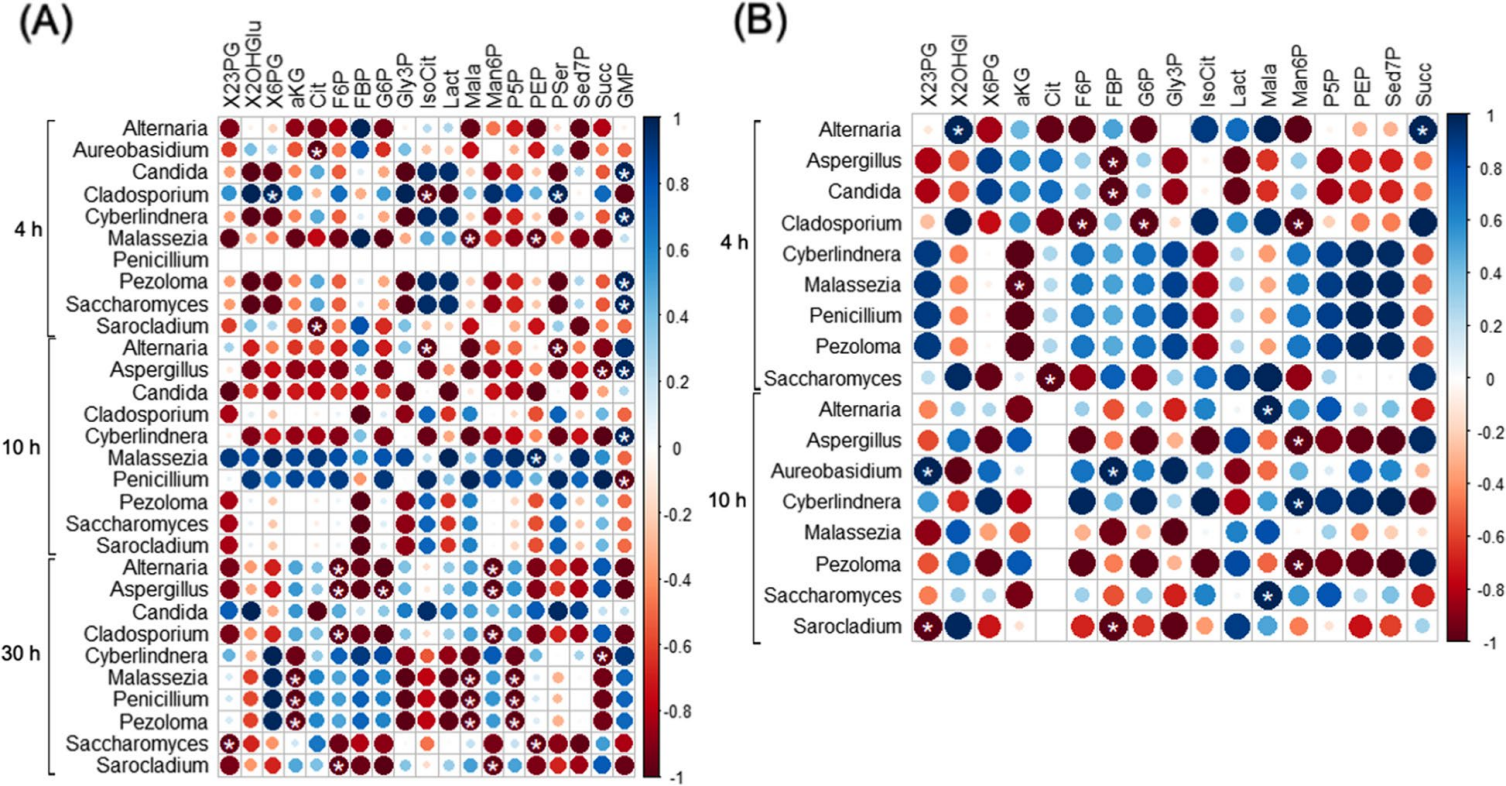
Correlation analysis of ^13^C-enriched fungi and ^13^C-enriched metabolites in females (**A**) and males (**B**). *Indicates significant positive and negative correlations (*p* < 0.05). (F6P, fructose-6-phosphate; FBP, fructose-1,6-bisphosphate; Gly-3P, glyceraldehyde-3-phosphate; 2/3-PG, 2/3-phosphoglycerate; PEP, phosphoenolpyruvate; Lact, lactate; Cit, citrate; IsoCit, isocitrate; a-KG, α-ketoglutarate; Succ, succinate; Mala, malate; Man6P, mannose-6-phosphate; G6P, glucose-6-phosphate; 6-PG, 6-phosphogluconate; P5P, pyridoxal-5-phosphate; Sed7P, sedoheptulose-7-phosphate; P-Ser, phosphoserine). Results are representative of three biological replicates per mosquito sex and time following fructose ingestion

Fungal communities were identified in the light and heavy DNA fractions of the mosquito guts collected directly at emergence (initial gut mycobiota) and after 4 h, 10 h, or 30 h of ^13^C-fructose ingestion. At the family level, the mycobiota gut composition varied according to the time of ^13^C-fructose ingestion and the sex of the mosquitoes (Fig. 5). For example, the Davidiellaceae family was dominant in females in all contexts, whereas in males, it was scarce in the initial mycobiota and dominant after ^13^C-fructose ingestion. The abundance of the Saccharomycetaceae and Wallemiaceae families decreased after ^13^C-fructose ingestion in both females and males. In contrast, the abundances of Trichocomaceae and Dothioraceae in females and Diatrypaceae in males increased as soon as ^13^C-fructose was ingested but decreased after 10 h for Diatrypaceae and Dothioraceae. A more detailed analysis at the genus level based on the estimation of a DNA-enrichment factor (EF) revealed differences in the enrichment of the different genera following the time of ^13^C-fructose assimilation in both sexes. In females, some genera (i) were enriched at 4 h (*Aureobasidium* and *Malassezia*), (ii) showed an increasing enrichment from 4 to 30 h (*Cyberlindnera*), (iii) were not detected at 4 h but were highly enriched at 10 h, with decreasing enrichment at 30 h (*Aspergillus* and *Saccharomyces*), or (iv) were enriched at 30 h (*Penicillium*, *Sarocladium*, *Pezoloma*, and *Alternaria*) (Fig. 6A). In males, some genera (i) similar to those in females showed an increasing enrichment over time (*Cyberlindnera*, *Alternaria*, *Aspergillus*, *Saccharomyces*, and *Malassezia*) or (ii) were enriched at 4 h (*Candida*) and 10 h (*Aureobasidium*). *Cladosporium* was the only genus enriched across all time points in both females and males (Fig. 6A). The correlation analysis between the ^13^C-enriched fungal taxa and fructose-derived metabolites indicated some patterns of specific associations between metabolites and fungi over time and according to the mosquito sex (Fig. 7). For instance, in females at the earlier time points (4 h and 10 h), GMP was positively correlated with *Candida*, *Cyberlindnera*, *Pezoloma*, *Saccharomyces*, *Aspergillus*, and *Cyberlindnera*. Moreover, *Cladosporium* was correlated with 6-PG and the P-serin (PSer) at 4 h, and *Malassezia* was correlated with phosphoenolpyruvate (PEP) at 10 h. In males at 4 h, *Alternaria* was positively correlated with 2-hydroxyglutarate (2-OHGlu) and succinate (Succ), while at 10 h, *Alternaria* and *Saccharomyces* were positively correlated with malate (Mala), *Cyberlindnera* with mannose-6-phosphate (Man6P), and *Aureobasidium* with 2,3-bisphosphoglycerate (2,3PG) and fructose-bis-phosphate (FBP).

## Discussion

Sugar digestion is mainly carried out by insect α-glucosidases [7]. However, the interplay between the insect gut microbiota and host sugar metabolism is also important to consider as the mosquito gut is inhabited by a diverse and metabolically active microbial ecosystem. This study provides the first joint study of microbiota and metabolome to evaluate host-microbiome interactions in mosquitoes. Metabolomics coupled with ^13^C isotope-labeled fructose has been used to track which major metabolic pathways are active after fructose assimilation in male and female *Ae. albopictus* mosquitoes and to target mycobiota dynamics associated with fructose metabolism. Bacteria are also important contributors of fructose metabolism, as previously shown [12]. Direct cross talk also operates through the microbiota. Microbes frequently interact with each other, but competitive association between microorganisms also exists, as revealed by recent findings in the human gut [42]. In this study, we focused on fungi, but we cannot rule out that, within gut mosquito, fungi compete for resources and/or provide some nutrients that can be directly used by bacteria. To date, very few information is available on sugar metabolism by insect microbiota, and few studies have characterized the fermentative metabolism of gut microorganisms. Microbiota carbohydrate metabolism leads to the production of acetate and related products, which can be directly used by the insect host [43]. It was also suggested that fructose-feeding insects host high numbers of fructophilic lactic acid bacteria (FLAB) [44, 45]. FLAB are distinguished from other lactic acid bacteria by the presence of specific biochemical characteristics allowing them to ferment fructose [46]. Interestingly, recent studies highlighted that some yeasts preferred fructose over glucose as carbon source, which is known as fructophily. This preference for fructose is explained by the fact that it can be converted directly to mannitol in a reaction with impact on redox balance [47]. Cabral et al. (2015) showed that this fructophilic character was ubiquitous in the *Zygosaccharomyces* and *Starmerella* clades [48]. The acquisition of fructophily would be concurrent with a wider remodeling of central carbon metabolism resulting by the loss of alcoholic fermentation in an ancestor of these two clades and the acquisition from bacteria of an enzyme required for sucrose assimilation [49]. *Hanseniaspora guilliermondii* is the only species preferring fructose, while it does not belong to the above clades. Even if the genus *Hanseniaspora* was not detected in field populations of *Ae. albopictus*, this genus was already shown to be associated with the midgut of adult *Aedes triseriatus* and *Anopheles stephensi* mosquitoes and merits further investigation [13].

### Fructose metabolism is driven by mosquito sex

Previous studies have shown differences in fat storage in mosquitoes according to the sex [50]. Moreover, it was also reported that the underlying difference in metabolic requirements between males and females is reflected in their preference for different nectar sources. It has been shown that diapausing females preferentially feed on flower nectars that are rich in monosaccharides (glucose and fructose), while males that do not undergo diapause tend to preferentially feed on flower nectars with high sucrose concentrations; hence, they do not compete against females for the same carbon source [51]. This study is the first to highlight differences in metabolic pathways between male and female guts after fructose ingestion, which probably indicates different modes of central carbon and metabolism regulation according to mosquito trophic traits. Previous studies on several mosquito species have reported that many factors such as body size, activity level, feeding, or digestion status may affect metabolic rate [6, 52–54]. It was previously shown that sex differences in metabolic rate could result from dimorphism in the performance of energetically demanding activities. Like most insects, mosquitoes show sexual dimorphism with sex-specific physical, physiological, and behavioral traits related to mosquito reproduction [55, 56]. As flight activity and mate searching differ according to male and female mosquitoes, we could expect differences in metabolic rates between both sexes. Differences in metabolic rates between both sexes could thus reflect the different modes of central carbon and metabolism regulation we highlighted in this study.

We observed earlier an increased alpha-ketoglutarate incorporation in males compared to females. This metabolite is a TCA cycle intermediate and thus an important metabolite required for energy production and cell growth. Interestingly, this metabolite was found to increase life span in the nematode *Caenorhabditis elegans* in a concentration-dependent way [57]. In *D. melanogaster* flies, it was suggested that high concentrations of a-KG are more toxic for male than for female ones, with males exhibiting shorter mean life span but unchanged maximum life span compared to females [58]. Whether the same effect occurs in mosquitoes is unknown. If so, the result of apparently earlier and increased a-KG incorporation in males would mean that male mosquitoes would metabolize more quickly a-KG to avoid a toxic effect at high concentrations.

We showed that lactate, the metabolic end product of glycolysis, which is involved in the production of energy and is recycled under anaerobic conditions to reenter the glycolysis process [59], was more significantly enriched in females than in males. As lactate can serve as a precursor for carbohydrate synthesis via gluconeogenesis, it is likely that female mosquitoes may accumulate more lactate to produce carbohydrates. We made this assumption as both sexes exhibit different ecological behaviors in terms of nutritional and dispersal capabilities [11]. In comparison to males, female mosquitoes disperse farther and develop host-seeking behaviors to find a suitable blood meal, followed by the selection of oviposition site [6, 60]. This trait has already been observed in many mosquito species, especially during starvation or migratory flight as well as in insect species whose diet is low in carbohydrates, such as blood-sucking insects [61].

It seems that guts contribute less to fructose metabolism in comparison with the whole mosquitoes, suggesting that fructose metabolism can occur in other parts of the body. This result contrasts with previous observations made in *An. aquasilis* suggesting that sugar digestion occurs essentially in the gut [7]. The crop is the primary storage organ for sugars before it migrates into the gut. Recent evidence of a rich and diverse microbial community in this organ in *Ae. albopictus* raises questions about the involvement of crop microbiota in fructose metabolism [62]. As previously shown in *Ae. aegypti* mosquitoes, ten amino acids are indispensable for larval nutrition and are sufficient for an adult female to produce mature, viable eggs [37]. In our study, 9 out of the 10 essential amino acids were labeled in the entire mosquito, while no labelling was detected in the gut. This finding may indicate that (i) fructose and its metabolites do not serve as precursors for amino acid production; (ii) no amino acid synthesis occurred in the guts, suggesting that the gut mycobiota is not involved in amino acid synthesis; and (iii) precursor compounds (fructose and its metabolites) were transferred from the gut to the site of amino acids production. However, we cannot exclude that the absence of amino acids in the guts is not due to the detection limit of the approach used.

### Dynamics of mycobiota actively involved in fructose metabolism

**Fig. 8:**
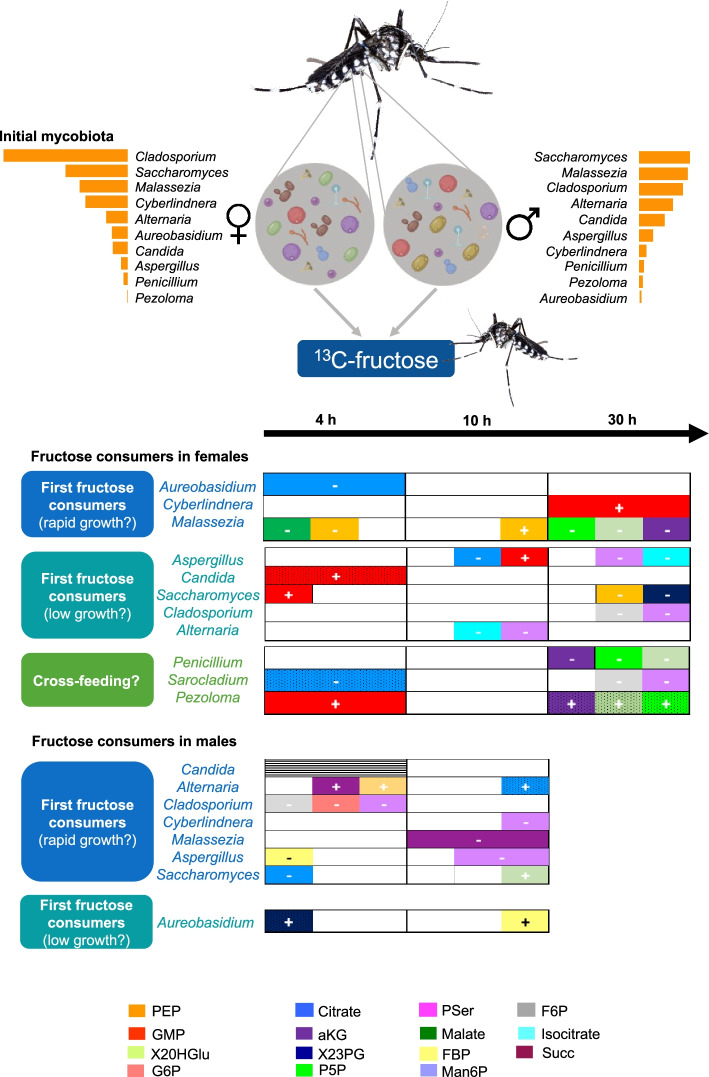
^13^C-fructose assimilation into the gut mycobiota of *Aedes albopictus* mosquitoes. The fungal genera identified as ^13^C enriched are indicated and positively (+) or negatively (−) at each time point of ^13^C-fructose ingestion (4 h, 10 h, and 30 h). The size of the bars is proportional to the value of the enrichment factor for each genus, except for the orange bars of the initial mycobiota that are proportional to the relative abundance of enriched OTUs in each genus. The colors of the bars indicate the metabolites that are correlated to the presence of each fungal genus and are described in the legend. Dot pattern indicated nonenriched fungal genera but correlated (+ or −) with metabolites. Hatched patterns correspond to fungal genera enriched but not correlated with identified metabolites

Primary fructose consumers identified in this study (closely related or similar species) were shown to (i) harbor enzymatic machinery involved in fructose metabolism (metabolic biochemical fructose pathways referenced in the Kyoto Encyclopedia of Genes and Genomes (KEGG) pathway database), and (ii) other species were already described for their ability to assimilate fructose [63, 64]. These observations support our findings and highlight the contribution of these fungi to fructose metabolism. In addition, differences in ^13^C-fructose assimilation by fungi were observed over time. Interestingly, the analysis of the mycobiota in non-sugar-fed mosquitoes indicated that all metabolically active fungi except for the genus *Sarocladium* were initially present in both females and males (summarized in Fig. 8). This evidence suggests that similar metabolically active fungal genera were identified in males and females; their dynamics was different over time. *Aureobasidium* was the most active genus at 4 h in females, whereas in males, it was active only after 10 h. The decrease in its abundance at 10 h in females was accompanied by a shift in the active mycobiota composition resulting in an increase in *Aspergillus*, *Saccharomyces*, and *Candida*. Conversely, in males, the increase in *Aureobasidium* was associated with a decrease in the abundance of *Candida*. This suggests that *Aureobasidium* could be a primary consumer of fructose in both sexes that does not persist over time due to competition among fungal communities. *Aureobasidium*, an ubiquitous genus found in water, soil, and plants, has been previously identified as a part of the core mycobiota of *Ae. albopictus*, as well as in plant nectar, the primary mosquitoes food source [65]. This genus is able to synthetize antifungal compounds that are toxic to some species of *Candida* and *Aspergillus*, known antagonists of *Aureobasidium* [66]. Interestingly, *Alternaria,* known to antagonize *Aureobasidium*, was identified only in the absence of this fungus [67]. This finding raises questions about competition for fructose uptake among mosquito mycobiota. *Aureobasidium*, by consuming fructose much faster in females, would compete to preferentially colonize gut niches at early times. Since the synthesis of antifungal compounds by *Aureobasidium* is induced by glucose and repressed by sucrose [68], the sugar composition of floral nectar may have important implications for fungal interactions within mosquitoes. In females, the identification of fungi enriched only at late times after fructose ingestion (e.g., *Penicillium* and *Pezoloma*, after 24 h [12] and *Sarocladium* after 30 h) indicates that either these species may be slowly growing fungi or that cross-feeding interactions may occur (i.e., species feed on products derived from fructose metabolism by other species). Finally, enrichment of these fungi was also correlated with a decrease in the enrichment of *Cyberlindnera* and *Candida*. Interestingly, the species *Sarocladium kiliense* was shown to degrade cellulose in the termite *Reticulitermes santonensis* [69]. Thus, this fungus is of interest due to its ability to metabolize various sugars. An increase in mycobiota activity was observed at 30 h. As mosquitoes had access to the fructose solution throughout the experiment, it is possible that the mosquito may have fed more than once between 10 and 30 h. However, this increase in activity could also be explained by cross-feeding phenomena, which are frequently observed across multiple kingdoms in the gut microbiome. Microbial communities can organize into food chains, with one species providing intermediate metabolites (released from microbial degradation or as waste products of metabolism) that will be used by the host or its associated microbiota [68]. To date, cross-feeding interactions have been well described in bacteria but less in fungi [70, 71]. In *Drosophila melanogaster*, *Lactobacillus plantarum* supplies lactate to *Acetobacter pomorum* which uses it to produce amino acids that are essential for *L*. *plantarum* growth [72]. However, in mosquitoes, such microbiota-microbiota interactions have not yet been described, and achieving a better understanding of the complex mosquito microbiota networks is proposed as a challenge for future mosquito microbiome research [73].

### Active fungal species correlate with ^13^C-enriched fructose metabolites

The identification of generalist and/or specialist fungi among fructose consumers in males and females could partly explain the observed differences in fructose metabolism between sexes. Consistent with this hypothesis, a sex effect was already corroborated in mosquito bacterial and fungal composition following 24 h of fructose ingestion [12]. Moreover, the enrichment of some metabolites, especially those involved in glycolysis and the TCA cycle, positively correlated with the presence of some fungal genera, and specific correlations were highlighted over time according to mosquito sex. In females, the two genera identified as primary fructose consumers that likely exhibited rapid growth (*Cyberlindnera* and *Malassezia*) were correlated respectively with GMP and PEP; among primary fructose consumers exhibiting slow growth, only *Aspergillus* was correlated with GMP. In males, three genera identified as primary fructose consumers that likely probably rapid growth (*Alternaria*, *Cyberlindnera*, and *Saccharomyces*) correlated with various enriched metabolites such as 2OHGlu, succinate, malate, and Man6P. *Aureobasidium*, the only fungal genus identified as a primary fructose consumer with low growth, was correlated with 2,3PG and FBP. Fungi are able to use diverse sources of carbohydrates to produce metabolites and essential amino acids [74]. The supply of essential amino acids from the gut microbiota to the host has already been demonstrated in many insects but has scarcely been explored in mosquitoes [75]. For example, in the Asian long-horned beetle, the gut microbiome, which include bacteria, filamentous fungi, and yeasts, was shown to be involved in supplying essential amino acids to the host [76]. The presence of complete biosynthetic pathways for most of the enriched amino acids (http://www.kegg.jp/) in some members of the active mosquito mycobiota supports the hypothesis that they could provide essential amino acids to the mosquito. Interestingly, *Aureobasidium pullulans*, one of the most enriched fungi identified in our study, was shown to synthesize leucine, isoleucine, lysine, valine, and phenylalanine in the eastern subterranean termite [77]. However, as previously mentioned [78], it is difficult to state with certainty which specific member is responsible for essential amino acid supply, often because microbial communities do not remain static over time. Interestingly, a recent study suggested that the mosquito microbiota may be involved in vitamin B biosynthesis and thus may provide a metabolic contribution to mosquito development [79].

## Conclusions

Taken together, our data highlight the major fructose-related metabolic pathways in the mosquito holobiont and the dynamics of the key fungal genera involved in fructose metabolism. The gut mycobiota represents a considerable source of diverse microbial activity. This study opens new avenues for the investigation of trophic interactions in mosquitoes and the interplay between microbial fructose metabolism and the mosquito host. In the future, ^13^C-metabolomics analyses of axenic and gnotobiotic mosquitoes with different microbial assemblages as well as more knowledge of the metabolic capacities of naturally mosquito-associated fungal species could help to reveal cross-feeding and metabolic interactions. Importantly, these multi-partner interactions and their modulation by the environment deserve more attention as they could serve as interesting targets for the development of alternative and specific mosquito control methods.

## Supplementary Information

**Additional file 1: Table S1**. Measure of 13C-fructose levels in guts and whole mosquito bodies for each sex at each time point following fructose ingestion.

**Additional file 2: Table S2**. Statistical analysis to evaluate sex differences for each isotopologue retrieved from the main metabolites presented in Fig. 2. (XLS 36 kb)

**Additional file 3: Table S3**. 13C enrichement (δ ‰ ) of heavy DNA fractions calculated from females and males at 4h, 10h and 30h following fructose ingestion. Results are representative of three biological replicates per mosquito sex and time points (mean ± SD). ND=not determined. (XLS 8 kb)

## Data Availability

All fastQ files were deposited at EMBL European Nucleotide Archive under the accession project number PRJEB45239 (https://www.ebi.ac.uk/ena). All analysis codes and parameters were deposited in the following site: https://github.com/morganeguegan/Mosquito-sex-and-active-mycobiota-are-important-contributors-of-fructose-metabolism-in-the-mosquito.git.

## References

[1] WHO. Vector-borne diseases, 2022 [Internet]. 2022. Available from: https://www.who.int/news-room/fact-sheets/detail/vector-borne-diseases.

[2] Foster WA (1995). Mosquito sugar feeding and reproductive energetics. Annu Rev Entomol.

[3] Nayar JK, Van Handel E (1971). The fuel for sustained mosquito flight. J Insect Physiol.

[4] Wang M, An Y, Gao L, Dong S, Zhou X, Feng Y (2021). Glucose-mediated proliferation of a gut commensal bacterium promotes *Plasmodium* infection by increasing mosquito midgut pH. Cell Rep.

[5] Marinotti O, de Brito M, Moreira CK (1996). Apyrase and α-glucosidase in the salivary glands of *Aedes albopictus*. Comp Biochem Physiol Part B Biochem Mol Biol.

[6] Clements AN (1992). The biology of mosquitoes. Vol. I. Development, nutrition and reproduction.

[7] Souza-Neto JA, Machado FP, Lima JB, Valle D, Ribolla PEM (2007). Sugar digestion in mosquitoes: identification and characterization of three midgut α-glucosidases of the neo-tropical malaria vector *Anopheles aquasalis* (Diptera: Culicidae). Comp Biochem Physiol Part A Mol Integr Physiol.

[8] Horvath TD, Dagan S, Scaraffia PY. Unraveling mosquito metabolism with mass spectrometry-based metabolomics. Trends in Parasitology. 2021; In press. Available from: https://www.cell.com/trends/parasitology/abstract/S1471-4922(21)00078-7. Cited 2021 May 24. 10.1016/j.pt.2021.03.010 PMC8282712 33896683

[9] Horvath TD, Dagan S, Lorenzi PL, Hawke DH, Scaraffia PY (2018). Positional stable isotope tracer analysis reveals carbon routes during ammonia metabolism of *Aedes aegypti* mosquitoes. FASEB J.

[10] Guégan M, Zouache K, Démichel C, Minard G, Tran Van V, Potier P (2018). The mosquito holobiont: fresh insight into mosquito-microbiota interactions. Microbiome..

[11] Minard G, Mavingui P, Moro CV (2013). Diversity and function of bacterial microbiota in the mosquito holobiont. Parasit Vectors.

[12] Guégan M, Van VT, Martin E, Minard G, Tran F-H, Fel B (2020). Who is eating fructose within the *Aedes albopictus* gut microbiota?. Environ Microbiol.

[13] Malassigné S, Valiente Moro C, Luis P (2020). Mosquito mycobiota: an overview of non-entomopathogenic fungal interactions. Pathogens.

[14] Steyn A, Roets F, Botha A (2016). Yeasts associated with *Culex pipiens* and *Culex theileri* mosquito larvae and the effect of selected yeast strains on the ontogeny of *Culex pipiens*. Microb Ecol.

[15] Souza RS, Virginio F, Riback TIS, Suesdek L, Barufi JB, Genta FA (2019). Microorganism-based larval diets affect mosquito development, size and nutritional reserves in the yellow fever mosquito *Aedes aegypti* (Diptera: Culicidae). Front Physiol.

[16] Valzania L, Martinson VG, Harrison RE, Boyd BM, Coon KL, Brown MR (2018). Both living bacteria and eukaryotes in the mosquito gut promote growth of larvae. PLOS Neglected Tropical Diseases. Public Library of. Science.

[17] Valzania L, Coon KL, Vogel KJ, Brown MR, Strand MR (2018). Hypoxia-induced transcription factor signaling is essential for larval growth of the mosquito *Aedes aegypti*. Proc Natl Acad Sci U S A.

[18] Correa MA, Matusovsky B, Brackney DE, Steven B (2018). Generation of axenic *Aedes aegypti* demonstrate live bacteria are not required for mosquito development. Nat Commun.

[19] Álvarez-Pérez S, Lievens B, Fukami T (2019). Yeast–bacterium interactions: the next frontier in nectar research. Trends Plant Sci.

[20] Tawidian P, Rhodes VL, Michel K (2019). Mosquito-fungus interactions and antifungal immunity. Insect Biochem Mol Biol.

[21] Guégan M, Minard G, Tran F-H, Tran Van V, Dubost A, Valiente MC (2018). Short-term impacts of anthropogenic stressors on *Aedes albopictus* mosquito vector microbiota. FEMS Microbiol Ecol.

[22] Minard G, Tran FH, Van VT, Goubert C, Bellet C, Lambert G (2015). French invasive Asian tiger mosquito populations harbor reduced bacterial microbiota and genetic diversity compared to Vietnamese autochthonous relatives. Front Microbiol.

[23] Haichar FEZ, Achouak W, Christen R, Heulin T, Marol C, Marais M-F (2007). Identification of cellulolytic bacteria in soil by stable isotope probing. Environ Microbiol.

[24] Rozier C, Erban A, Hamzaoui J, Prigent-Combaret C, Comte G, Kopka J (2016). Xylem sap metabolite profile changes during phytostimulation of maize by the plant growth-promoting rhizobacterium, *Azospirillum lipoferum* CRT1. Metabolomics..

[25] Kiefer P, Schmitt U, Vorholt JA (2013). eMZed: an open source framework in Python for rapid and interactive development of LC/MS data analysis workflows. Bioinformatics..

[26] Millard P, Delépine B, Guionnet M, Heuillet M, Bellvert F, Létisse F. IsoCor: isotope correction for high-resolution MS labeling experiments. Bioinformatics. 2019;35(21):4484–7. 10.1093/bioinformatics/btz209 30903185

[27] Luis P, Vallon L, Tran F-H, Hugoni M, Tran-Van V, Mavingui P (2019). *Aedes albopictus* mosquitoes host a locally structured mycobiota with evidence of reduced fungal diversity in invasive populations. Fungal Ecol.

[28] Escudie F, Auer L, Bernard M, Cauquil L, Vidal K, Maman S (2016). FROGS: find rapidly OTU with galaxy solution. F1000Research..

[29] Schloss PD, Westcott SL, Ryabin T, Hall JR, Hartmann M, Hollister EB (2009). Introducing mothur: open-source, platform-independent, community-supported software for describing and comparing microbial communities. Appl Environ Microbiol.

[30] Wang Q, Garrity GM, Tiedje JM, Cole JR (2007). Naive Bayesian classifier for rapid assignment of rRNA sequences into the new bacterial taxonomy. Appl Environ Microbiol.

[31] Kõljalg U, Nilsson RH, Abarenkov K, Tedersoo L, Taylor AFS, Bahram M (2013). Towards a unified paradigm for sequence-based identification of fungi. Mol Ecol.

[32] R Core Team 2018 (2018). R a language and environment for statistical computing.

[33] McMurdie PJ, Holmes S (2013). phyloseq: an R package for reproducible interactive analysis and graphics of microbiome census data. PLoS One.

[34] Oksanen J. vegan: Community ecology package. R package version 2.0-2. 2011. http://CRANR-project.org/package=vegan. Available from: https://ci.nii.ac.jp/naid/20001323876/. Cited 2018 Apr 20.

[35] Wickham H (2016). ggplot2: elegant graphics for data analysis.

[36] Paradis E, Claude J, Strimmer K (2004). APE: analyses of phylogenetics and evolution in R language. Bioinformatics..

[37] Slowikowski K (2018). ggrepel: automatically position non-overlapping text labels with “ggplot2”.

[38] Wickham H (2011). The split-apply-combine strategy for data analysis. J Stat Softw.

[39] Wickham H, François R, Henry L, Müller K. dplyr: A Grammar of Data Manipulation. 2022. https://dplyr.tidyverse.org, https://github.com/tidyverse/dplyr.

[40] Wei T, Simko V, Levy M, Xie Y, Jin Y, Zemla J. corrplot: visualization of a correlation matrix. https://CRAN.R-project.org/package=corrplot [Internet]. 2017. Available from: https://CRAN.R-project.org/package=corrplot. Cited 2021 Mar 21.

[41] Hünninghaus M, Dibbern D, Kramer S, Koller R, Pausch J, Schloter-Hai B (2019). Disentangling carbon flow across microbial kingdoms in the rhizosphere of maize. Soil Biol Biochem.

[42] Zuo T, Kamm MA, Colombel J-F, Ng SC (2018). Urbanization and the gut microbiota in health and inflammatory bowel disease. Nat Rev Gastroenterol Hepatol.

[43] Ankrah NYD, Douglas AE (2018). Nutrient factories: metabolic function of beneficial microorganisms associated with insects. Environ Microbiol.

[44] Endo A, Salminen S (2013). Honeybees and beehives are rich sources for fructophilic lactic acid bacteria. Syst Appl Microbiol.

[45] Neveling DP, Endo A, Dicks LMT (2012). Fructophilic Lactobacillus kunkeei and Lactobacillus brevis isolated from fresh flowers, bees and bee-hives. Curr Microbiol.

[46] Endo A, Maeno S, Tanizawa Y, Kneifel W, Arita M, Dicks L (2018). Fructophilic lactic acid bacteria, a unique group of fructose-fermenting microbes. Appl Environ Microbiol.

[47] Gonçalves C, Ferreira C, Gonçalves LG, Turner DL, Leandro MJ, Salema-Oom M (2019). A new pathway for mannitol metabolism in yeasts suggests a link to the evolution of alcoholic fermentation. Front Microbiol.

[48] Cabral S, Prista C, Loureiro-Dias MC, Leandro MJ (2015). Occurrence of FFZ genes in yeasts and correlation with fructophilic behaviour. Microbiology (Reading).

[49] Gonçalves C, Wisecaver JH, Kominek J, Oom MS, Leandro MJ, Shen X-X (2018). Evidence for loss and reacquisition of alcoholic fermentation in a fructophilic yeast lineage. Elife..

[50] van Handel E (1984). Metabolism of nutrients in the adult mosquito. Mosquito News.

[51] Chang J, Singh J, Kim S, Hockaday WC, Sim C, Kim SJ (2016). Solid-state NMR reveals differential carbohydrate utilization in diapausing *Culex pipiens*. Sci Rep.

[52] Gray EM, Bradley TJ (2003). Metabolic rate in *Culex tarsalis* (Diptera: Culicidae): age, size, activity, and feeding effects. J Med Entomol.

[53] Gray EM, Bradley TJ (2006). Malarial infection in *Aedes aegypti*: effects on feeding, fecundity, and metabolic rate. Parasitology.

[54] Burggren W, Souder BM, Ho DH (2017). Metabolic rate and hypoxia tolerance are affected by group interactions and sex in the fruit fly (*Drosophila melanogaster*): new data and a literature survey. Biol Open.

[55] Wormington JD, Juliano SA (2014). Sexually dimorphic body size and development time plasticity in *Aedes* mosquitoes (Diptera: Culicidae). Evol Ecol Res.

[56] Duman-Scheel M, Syed Z (2015). Developmental neurogenetics of sexual dimorphism in *Aedes aegypti*. Front Ecol Evol.

[57] Chin RM, Fu X, Pai MY, Vergnes L, Hwang H, Deng G (2014). The metabolite α-ketoglutarate extends lifespan by inhibiting ATP synthase and TOR. Nature..

[58] Lylyk MP, Bayliak MM, Shmihel V, Storey JM, Storey KB, Lushchak VI (2018). Effects of alpha-ketoglutarate on lifespan and functional aging of *Drosophila melanogaster* flies. Ukr Biochem J.

[59] Hall MM, Rajasekaran S, Thomsen TW, Peterson AR (2016). Lactate: friend or foe. PM R.

[60] Becker N, Petric D, Zgomba M, Boase C, Madon M, Dahl C (2010). Mosquitoes and their control.

[61] Candy DJ, Becker A, Wegener G (1997). Coordination and Integration of metabolism in insect flight. Comp Biochem Physiol Part B Biochem Mol Biol.

[62] Guégan M, Martin E, Valiente MC (2020). Comparative analysis of the bacterial and fungal communities in the gut and the crop of *Aedes albopictus* mosquitoes: a preliminary study. Pathogens.

[63] Sheng L, Tong Q, Ma M (2016). Why sucrose is the most suitable substrate for pullulan fermentation by *Aureobasidium pullulans* CGMCC1234?. Enzym Microb Technol.

[64] Endoh R, Horiyama M, Ohkuma M (2021). D-Fructose assimilation and fermentation by yeasts belonging to Saccharomycetes: rediscovery of universal phenotypes and elucidation of fructophilic behaviors in *Ambrosiozyma platypodis* and *Cyberlindnera americana*. Microorganisms..

[65] Alvarez-Pérez S, Herrera CM (2013). Composition, richness and nonrandom assembly of culturable bacterial-microfungal communities in floral nectar of Mediterranean plants. FEMS Microbiol Ecol.

[66] Prasongsuk S, Ployngam S, Wacharasindhu S, Lotrakul P, Punnapayak H (2013). Effects of sugar and amino acid supplementation on *Aureobasidium pullulans* NRRL 58536 antifungal activity against four *Aspergillus* species. Appl Microbiol Biotechnol.

[67] Prendes LP, Merín MG, Fontana AR, Bottini RA, Ramirez ML, Morata de Ambrosini VI (2018). Isolation, identification and selection of antagonistic yeast against *Alternaria alternata* infection and tenuazonic acid production in wine grapes from Argentina. Int J Food Microbiol.

[68] Tarayre C, Bauwens J, Brasseur C, Mattéotti C, Millet C, Guiot PA (2015). Isolation and cultivation of xylanolytic and cellulolytic *Sarocladium kiliense* and *Trichoderma virens* from the gut of the termite *Reticulitermes santonensis*. Environ Sci Pollut Res Int.

[69] Koropatkin NM, Cameron EA, Martens EC (2012). How glycan metabolism shapes the human gut microbiota. Nat Rev Microbiol.

[70] D’Souza G, Shitut S, Preussger D, Yousif G, Waschina S, Kost C (2018). Ecology and evolution of metabolic cross-feeding interactions in bacteria. Nat Prod Rep.

[71] Seth EC, Taga ME (2014). Nutrient cross-feeding in the microbial world. Front Microbiol.

[72] Henriques SF, Dhakan DB, Serra L, et al. Metabolic cross-feeding in imbalanced diets allows gut microbes to improve reproduction and alter host behaviour. Nat Commun. 2020;11:4236. 10.1038/s41467-020-18049-9 PMC7447780 32843654

[73] Dada N, Jupatanakul N, Minard G, Short SM, Akorli J, Villegas LM (2021). Considerations for mosquito microbiome research from the Mosquito Microbiome Consortium. Microbiome..

[74] Lewis DH (1991). Fungi and sugars — a suite of interactions. Mycol Res.

[75] Douglas AE (2009). The microbial dimension in insect nutritional ecology. Funct Ecol.

[76] Ayayee PA, Larsen T, Rosa C, Felton GW, Ferry JG, Hoover K (2016). Essential amino acid supplementation by gut microbes of a wood-feeding cerambycid. Environ Entomol.

[77] Ayayee PA, Jones SC, Sabree ZL (2015). Essential amino acid provisioning by termite-associated gut microbiota. PeerJ PrePrints.

[78] Engel P, Moran NA (2013). The gut microbiota of insects – diversity in structure and function. FEMS Microbiol Rev.

[79] Romoli O, Schönbeck JC, Hapfelmeier S, Gendrin M (2021). Production of germ-free mosquitoes via transient colonisation allows stage-specific investigation of host–microbiota interactions. Nat Commun.

